# History of Massage

**Published:** 1879-10

**Authors:** Douglas Graham

**Affiliations:** Boston, Mass.


### HISTORY OF MASSAGE.

By DOUGLAS GRAHAM, M. D., Boston, Mass.

In almost every city of the United States, and, indeed, of the whole civilized world, there may be found individuals claiming mysterious and magical powers of curing disease, setting bones, and relieving pain by the immediate application of their hands. Some of these boldly assert that their art is a gift from Heaven, due to some unknown power which they call magnetism; others designate it by some peculiar word ending with pathy or cure, and it is often astonishing how much credit they get for their supposed genius by the most learned people of the land outside of the medical profession. History informs us that rubbing, kneading, percussion, passive and resistive movements have been partly used, in some form or other, among savage and civilized nations, from the most ancient times. To express these various manœuvres collectively nearly all physicians who take any interest in the matter, foreign as well as American, seem satisfied with the French word *massage*, from the Greek *masso*, I knead or handle. But so little attention has this subject received, that Prof. Billroth, of Vienna. in 1875, and Dr. Wagner, of Friedberg, in 1876, stated that there were many physicians in Germany who had never heard of massage, and that it was then an every-day question as to what it meant, some even supposing that Dr. Mezger, of Amsterdam, was the originator of it. Therefore, it is no wonder that when an article appears on the subject it often gets the credit of being original by many who read it, but overlook the fact expressed so well in the words of Hippocrates, that "Medicine hath of old both a principle and a discovered track whereby in a long time many and fine discoveries have been discovered, and the rest will be discovered, if any one who is competent and knows what hath been discovered, start from these data on the search. But whoever, rejecting these, and despising all, shall undertake to search by a different track and in a different manner, and shall say that he hath discovered something, will be deceived himself and will deceive others." It is nothing unusual to see clear-headed individuals, uneducated as well as those of extensive learning, evolve from their inner consciousness, so to speak, something or other to meet the exigencies of a case, and then announce the same as a new discovery, when it had been put to similar purposes many times before. Such geniuses, if truly ignorant that their device had never before been heard of, deserve equally as great credit as the original inventors; but what very often detracts from the merit of the case is the fact that they did not stop to inquire whether any one else had ever used the same method or not. On the other hand, it is not always the one making use of something novel who claims originality, but frequently those who are not so well informed on the subject as he is who claim it for him. In the latter category almost every one has been delighted to find himself placed at some time or other, without any such intention on his part; and it is sometimes an agreeable excuse to let it pass, on the ground that it would not be dignified to inform the admirers of their ignorance. These remarks apply to massage and its advocates, as well as to other matters pertaining to medicine.

The earliest definite information concerning massage appears at about the beginning of the fourth century B. C., in the works of the great Greek physician, the Father of Medicine, Hippocrates. Though his notions, like those of the other ancients, were vague and incorrect with regard to the circulation of the blood (the important discovery of which by Harvey not taking place until more than 2,000 years later, A. D., 1628), yet he used rubbing in accordance with scientific principles, in such a way as to aid and not retard the circulation, as is shown by the word with which he defined the process, viz., *anatripsis*—literally, the art of rubbing up, not down. In this way doubtless, he had had experience in promoting resorption of effusions, as it is now well known that rubbing the limbs upward favors the return of the circulation, relieves blood-stasis, and makes room in the veins and lymphatics for the more speedy passage of morbid products. After the acute stage of an injury had elapsed Hippocrates applied massage as we learn from the following words: "The physician must be experienced in many things, but assuredly also in *anatripsis;* for things that have the same name have not always the same effects. For rubbing can bind a joint that is too loose, and loosen a joint that is too rigid, and the joint must be moved about," etc. Paradoxical as this may seem, yet I have witnessed the verification of it in two cases occurring in my own practice. They have already been reported in the New York *Medical Record* for August 11, 1877. The rationale of this statement however, is not so paradoxical; for by appropriate rubbing, kneading, and passive motion, atrophied muscles, tendons and ligaments would have their circulation accelerated and increased, and thus their nutrition and innervation improved so that they would grow larger and firmer, and as a natural consequence, a joint too lax from such causes would become stronger. In the other case, by the same means, involuntary tension of muscles, adhesions, effusions, and hyperplastic tissue may be removed, and thus a joint stiff from such causes be made suppler.

Furthermore, says old Hippocrates in his aphorisms: "Anatripsis can bind and loosen; can make flesh and cause parts to waste. Hard rubbing binds; soft rubbing loosens; much rubbing causes parts to waste; moderate rubbing makes them grow." These words seem like revelation to any one who has not had much experience in massage, and the literal fulfillment of them may be observed when the necessary previous conditions exist. Hard and soft, much and moderate are of course to be taken in a relative sense; for, what might be hard to one person might seem gentle to another.

Vigorous massage, as we might expect, makes soft and flabby muscles firmer. Gentle or moderate rubbing and manipulating loosens not only the abnormally tough and

*matted* condition of the skin and superficial fascia, but also the involuntary tenseness of the muscles, which, if looked for, may often be found not merely locally, but generally in overtasked and debilitated people. Such a state of these tissues would often seem to be a physical indication of too great mental tension, which the patient, like his muscles, is unable to relax. And here comes the necessity of a careful discrimination, which Hippocrates evidently appreciated, in using massage; for, if a patient in the condition described should receive such vigorous rubbing and kneading, as is so much advocated now-a-days, and which would even seem to be necessary to relax such tissues, his condition would in all probability be aggravated, for reflex action, and consequently still greater tension would be excited by the pressure of rough friction and manipulation upon terminal nerve-filaments which are already in a state of irritation. An admirable preliminary measure to the use of massage in such cases is the warm bath, for it is grateful and soothing to the patient, solicits the blood to the surface, softens the cuticle, and removes the epithelial debris, as well as relaxes the skin, and to some extent the tissues beneath it.

"Much rubbing causes parts to waste." I have seen people who had a normal quantity of adipose tissue loose much of it, to their detriment, from the excessive use of massage. But even this feature can sometimes be utilized to advantage in cases where fat is super-abundant, soft and flabby, with a want of tone and tension in the aroelar tissue. It is well known that tissues, when subjected to great and constant pressure, become attenuated and absorbed; but when acted upon by a less constant and slighter degree of pressure, they increase in thickness. "Moderate rubbing makes parts grow." This implies that the tissues to be rubbed are insufficiently nourished; and, that if they be immoderately rubbed are insufficiently nourished; and moreover, that if they be immoderately rubbed, their vitality will be lessened, their nervous irritability exhausted, and a state of congestion induced, highly unfavorable to their proper nourishment.

The ancients entertained sensible views with regard to the maintenance of health. "Not only Hippocrates, but all the physicians and philosophers of that period, knew no better means of strenghtening the vital principle and prolonging life than by moderation; the use of free and pure air, bathing, and, above all, by daily friction of the body and exercise. Rules and directions were laid down for giving violent and gentle motion to the body in a variety of ways—hence arose a particular art called the gymnastic; and the greatest philosophers and men of learning never forgot that the body and the soul ought to be exercised in due proportion. This art, to us almost unknown, of suiting exercise to the different constitutions, situations, and wants of man; of employing it, above all, as the means of keeping his internal nature in proper activity, and thereby rendering the causes of disease ineffectual, but also curing diseases which have already appeared, they, indeed, brought to an extraordinary degree of perfection." The gymnastics were divided into athletic, military, and medical. Herodicus, of Selivria, first proposed gymnastics for the cure of disease; and to such an extent, we are told, did he carry his ideas that he compelled his patients to exercise and suffer their bodies to be rubbed; and he had the good fortune to lenghthen for several years by this method the lives of so many enfeebled persons, that Plato reproached him for prolonging that existence of which they would have less and less enjoyment.

Nowhere, perhaps, does the wisdom of the ancients appear more strikingly full of truth and meaning than in some of their remarks about massage. The distinguished Roman physician, Celsus, who flourished at the beginning of the Christian era, said that "rubbing should sometimes be applied to the whole body, as when an invalid requires his symptoms to be replenished." "As rubbing is rightly applied after the cessation of an illness, so it must never be used during the increment of a fever, but, if possible, when the body shall have been wholly free from it." . . . . "A paralyzed limb is strengthened by being rubbed. If certain limbs only are rubbed, long and powerful rubbing may be used, for the whole body cannot soon be weakened through a part. But when weakness of the body needs this cure over its whole extent, it ought to be shorter and more gentle. . . . Chronic pains of the head are relieved by rubbing the head itself. But far more frequently, when one part is in pain another must be rubbed, and particularly when we desire to *draw matter* from the upper or middle part of the body, and therefore rub the extremities." This is not the place to illustrate these remarks with cases treated with the modern methods of massage, though it could easily be done. However, proceeding onward with the march of time we find that one hundred years later history furnishes a good example of the truth of the first two observations here quoted from Celsus. The health of the celebrated Roman advocate, Pliny, which was never very strong, had been shaken by a severe illness the preceding year A.D. 102. His life, he tells the emperor in one of his letters, had been in danger. He availed himself of a mode of treatment which it is presumed was much in vogue at that time. He procured the services of a medical practitioner who cured many of his patients by the process of rubbing and anointing. So much benefit did he derive from the remedy, that he asked the emperor to grant the physician who was either a Jew or Greek, the freedom of the city and the privilege of Roman citizenship. Cicero considered that he owed as much of his health to his anointer as he did to his physician.

In ancient times rubbing and anointing were much used in connection with the baths, the building for which were of great magnificence and luxury during the Roman empire, as their immense ruins yet existing testify. The Roman emperor Hadrian, seeing a veteran soldier one day rubbing himself against the marble of the public baths, asked him why he did so. The veteran answered, "I have no slave to rub me;" whereupon the emperor gave him two slaves and sufficient to maintain them. Another day several old men rubbed themselves against the wall in the emperor's presence, hoping for similar good fortune, when the shrewd Hadrian, perceiving their object, directed them to rub one another!

Martial (A.D. 100) undoubtedly refers to some kind of massage in the following lines:

> "Percurrit agili corpus arte tractatrix.
> Manumque doctum spargit omnibus membris.'

Galen (A.D. 170) recommended friction in a great number of diseases, generally as auxiliary to other means. At Pergamus he was appointed city physician to the school of gladiators. These were rubbed before and after their exercises and combats; before, in order to increase the elasticity and strength of their limbs; after, in order to stroke away the ecchymoses, relieve the pain of the bruises, and to rest and refresh them from their fatigue.

A great deal more might be said to show that the Greeks and Romans knew well the advantage of friction as a hygienic and as a therapeutic agent. But they were not the only members of the Aryan race who practiced rubbing. Strabo states that the Indians contemporary with Alexander, 326 B.C., esteemed friction highly. "In the way of exercise," he says, "they think most highly of rubbing; and they polish their bodies smooth with ebony staves and in other ways." "There are public baths in India which are associated with the practice of shampooing. The bather is extended upon a plank, and a vigorous attendant pours hot water over him, presses and bends the various parts of his body, cracks all his joints, and continues the operation of pouring, pulling and pressing for about half an hour. He then rubs him briskly by means of a hair brush with soap and perfumes, after which the subject is obliged by his fatigue to sleep a few hours, from which he awakes extremely refreshed. The women in India take a lively pleasure in being shampooed by their slaves; and Europeans, who enter upon the process with a sort of fear, describe the sensation which results as delightful and peculiar."

Much the same practices obtain among the Turks and Arabs. With the Russians flagellation and friction by means of a bundle of birch twigs are resorted to after the subject has been well parboiled in a vapor bath. A pailful of cold water is then dashed over him from head to foot, the effect of which is described as electrifying. After this he plunges into the snow, and thus tempers himself like steel to endure with impunity his vigorous climate. The Siberians and Laplanders also indulge in similar luxuries.

Having seen that massage has been practiced after a fashion by the Greeks, the Romans and the Hindoos, it is reasonable to suppose that it was also made use of by their common ancestors, the Aryans; so that its origin may well be spoken of as hoary with antiquity, or as some say, lost in the night of time. It may not be amiss, just here, to let history tell us who the Aryans were, and where their original place of abode was. The Aryan branch of the Caucassian race includes nearly all the past and present nations of Europe, and is that division to which we ourselves belong. The Aryans are described as being a fair-skinned, noble people, progressive, practical, and warlike, and it is said that they speedily subdued the country adjacent to them, and also the peninsula of India, 3000 years B. C. The original seat of the undivided Aryan stock was to the north-east of Persia, in the region of the Oxus and Jaxartes rivers. The water of the Oxus is said to have been extremely soft, so as to have made the skin of those who bathed in it glisten. Now, we can imagine that from the pleasant sensation of drawing the hand over the skin in admiration of the effect of the water of the Oxus upon it, may have arisen the practice of systematic friction among the Aryans.

So much for the history of massage in what might be called its period of invention. We will now briefly glance at what might be styled its period of renewal, in the sixteenth century, as but little of interest can be found worth mentioning till that sime. Ambrose Pare, in his works, which were published in 1575, states that friction was in great esteem in his time. He describes three kinds of friction, the gentle, the medium, and the rough, and the effects of each. In dislocations he recommends that the joint should be moved about this and that way—not violently—in order to resolve the effused fluids and extend the fibres of the muscles and of the ligaments, so as to facilitate the reduction. From this it is apparent that he knew the influence of passive motion in promoting absorption, the rationale of which has been so well studied of late by German physiologists.

Hoffman, in his *Dissertationes Physico-Medical*, 1708, says, "that exercise is the best medicine for the body." (It is not always applicable, however.) "We cannot imagine," he adds, "how much it is salutary and favorable to health; corporeal exercise excites the flow of the spirits and facilitates the excretions from the blood." He describes the passive, active, and mixed movements of the ancients, as well as the *apotherapeia* or perfect cure, meaning the last part of the ancient gymnastics, which consisted of friction, inunction, and bathing for the purpose of obviating fatigue and curing disease.

Alpinus, in his *Medicina Egyptia*, says that frictions are so extensively used among the Egyptians that no one retires from a bath without being rubbed. "For this purpose the person is extended, then he is malaxated (manipulated, kneaded) and pressed in divers manners upon the various parts of his body. Passive motion is then given to the different articulations. They are not satisfied with *masseeing*, flexing, and extending the articulations alone; they exercise the same pressures and the same frictions upon all the muscles." The effect of which is thus described by M. Savary: "Perfectly *masseed*, one feels completely regenerated, a feeling of extreme comfort pervades the whole system, the chest expands and we breathe with pleasure, the blood circulates with ease, and we have a sensation as freed from an enormous load; we experience a suppleness and lightness till then unknown. It seems as if we had just been born as if we truly lived for the first time. There is a lively feeling of existence which radiates to the extremities of the body, whilst this is given over to the most delightful sensations; the mind takes cognizance of these and enjoys the most agreeable thoughts; the imagination wanders over the universe which it adorns, sees everywhere smiling pictures, everywhere the image of happiness. If life were only a succession of ideas, the rapidity with which memory retraces them, the vigor with which the mind runs over the extended chain of them, would make one believe that in the hours of delicious calm which follows we live a great many years."

Shakespeare has not forgotten to mention rubbing and kneading:

> IAGO.—My lord has fallen into an epilepsy
> This is his second fit; he had one yesterday.
> CASSIO,—Rub him about the temples.
> IAGO.—No, forbear;
> The lethargy must have his quiet course.
>
> OTHELLO, iv. 1.
>
> AJAX.—I'll knead him; Ill make him supple.
>
> TROILUS AND CRESSIDA, ii. 3.

These lines indicate the empirical and the rational use of massage, though the latter was employed in a figurative sense. —*Medical Record.*

